# Vaccination-Induced Noncytolytic Effects in the Acute Phase of SHIV Infection

**DOI:** 10.1371/journal.pone.0015083

**Published:** 2010-11-30

**Authors:** Janka Petravic, Miles P. Davenport

**Affiliations:** Complex Systems in Biology Group, Centre for Vascular Research, University of New South Wales, Sydney, New South Wales, Australia; University of California San Francisco, United States of America

## Abstract

Many studies have shown that vaccines inducing CD8+ T cell responses can reduce viral loads and preserve CD4+ T cell numbers in monkey models of HIV infection. The mechanism of viral control by the vaccine-induced CD8+ T cells is usually assumed to be cytolysis of infected cells. However, in addition to cytolysis of infected cells, CD8+ T cells secrete a range of soluble factors that suppress viral replication. We have studied the dynamics of virus and CD4+ T cells in a successful vaccination-challenge model of SHIV infection. We find that better viral control in the acute phase of infection is associated with slower decay of peak viral load. Comparing viral and CD4+ T cell dynamics in acute infection, we find that a cytolytic mode of viral control with direct killing of infected cells is inconsistent with the observed trends. On the other hand, comparison of the predicted effects of noncytolytic CD8+ effector function with the experimental data shows that non-cytolytic control provides a better explanation of the experimental results. Our analysis suggests that vaccine-induced CD8+ T cells control SHIV infection by non-cytolytic means.

## Introduction

Virus-specific CD8+ T cells play an important role in control of HIV-1 infection in humans and simian immunodeficiency virus (SIV) infection in macaques [1], [2], [3], [4]. After T-cell receptor interaction with peptide/major histocompatibility class I (MHC-I) complexes, CD8+ T cells proliferate and express a variety of effector functions that inhibit viral replication. These include direct lysis of infected cells [5] and release of a range of cytokines [6], which may suppress production of new virions by infected cells, or chemokines [7] inhibiting viral entry into the host cells. Different types of CD8+ T-cell antiviral activity have been shown in vivo by FACS sorting of cells expressing different markers in blood and tissue samples taken from HIV patients and SIV-infected monkeys [8], and in a range of in vitro experiments [9], [10], [11]. There is evidence that multifunctionality of CD8+ T cells correlates with the level of viral control [8], [12], and that HIV non-progressors exhibit strong noncytolytic response [13]. It is therefore important to determine which type of CD8+ T cell effector function is the most important in HIV/SIV control in vivo. Several studies of SIV dynamics in CD8-depleted rhesus macaques have addressed this question [2], [14], [15]. They showed that the magnitude and rate of rise in viral load following CD8+ T cell depletion was too rapid to be explained by increased lifespan of infected cells [2], and that the decay of SIV under antiretroviral treatment in the chronic phase of infection is not altered in the absence of CD8+ T cells [14], [15]. Similarly, we have recently demonstrated that the decay rates of wild-type and escape mutant virus are similar in SHIV infected macaques, and thus the dynamics of immune escape are inconsistent with cytolytic control of wild-type virus [16]. These results indicate that direct killing of infected cells might not be the dominant means of viral control in the chronic phase of SIV/SHIV infection.

Simian-human immunodeficiency virus (SHIV) infection of rhesus macaques provides a model for studies of potential protective ability of vaccines against HIV-1, where a large number of vaccines have proved effective [17]. Our aim is to determine, from the dynamics of the early and acute SHIV infection, whether the early CD8+ T cell response, stimulated by vaccines that generate cell-mediated immunity, is predominantly cytolytic or noncytolytic in this animal model.

In a recent paper [18], we have shown that in CXCR4-tropic SHIV-infected monkeys vaccination significantly reduced peak viral load and increased the lowest CD4+ T cell count in the acute phase of infection. Although we demonstrated the decrease in virus replication in vaccinated animals, we did not identify the specific mechanism (i.e. the CD8+ T cell effector function) responsible for this outcome.

Here we investigated the relationship between the peak viral load and the decay rate of virus in order to determine if the improved virus control consistently corresponds to increased direct killing of infected cells, or is better explained as a consequence of increased noncytolytic effector functions. We found that lower viral peak was associated with a slower decay of virus after the peak. The viral peak and the decay rate were positively correlated across all animals.

Using a modeling approach to investigate the dynamics of virus and CD4+ T cells, we find that the kinetics of viral load and the loss of CD4+ T cells to infection are not consistent with a cytolytic mechanism of CD8+ T cells killing SHIV infected cells. However, if the mechanisms of CD8+ T cell control were non-cytolytic, or involved killing of infected cells in a ‘window period’ before they produced virus, then the modeled dynamics of viral and CD4+ T cells would be consistent with the experimental data. This suggests that vaccine-induced virus-specific CD8+ T cells in SHIV infection control virus using non-cytolytic mechanisms.

## Results

Vaccination against SIV and SHIV results in varying degrees of protection, depending on the type of vaccine, viral strain and animal model. The effect of improved viral control in early infection in vaccinated animals can be seen as the decrease in the peak plasma viral load and reduced loss of CD4+ T cells in peripheral blood. We have recently shown [18], [19] that there is a strong positive correlation between peak viral load and CD4+ T cell loss in the acute phase. Peak viral loads are consistently and significantly lower in vaccinated animals than in unvaccinated controls.

**Figure 1:**
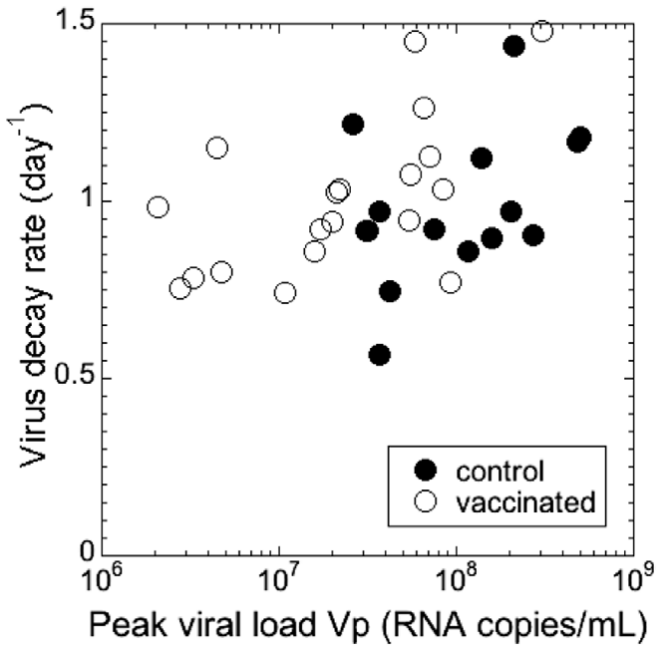
Correlation between peak and decay of viral load. Peak viral load and the decay rate of virus after the peak in SHIV_89.6P_ are positively correlated (Spearman *r* = 0.346 and *p* = 0.0418).

One limitation in comparing viral decay data in controls and vaccines is that the animals were vaccinated with a variety of regimes, some of which (like adenovirus 5) were very effective, while others (like Alum) were not. This can be seen in the overlap of viral peaks. In order to take into account the effectiveness of different vaccines or of the immune response in control animals, we investigated how the reduction in peak viral load affected viral decay. Figure 1 shows viral decay rates plotted against corresponding peak viral loads. We found a significant positive correlation between peak viral load and decay rate (Spearman *r* = 0.346 and *p* = 0.0418). That is, a more effective immune response, which led to better control of peak viremia, was actually associated with slower decay of virus after the peak.

### The dynamics of cytolysis

**Figure 2:**
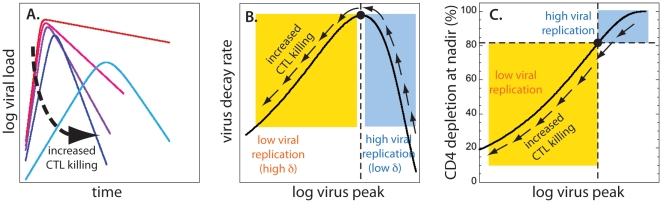
Cytolytic immune response. For cytolytic immune response, the correlation between peak viral load and the decay of virus can be positive or negative, depending on the level of CD4+ T cell depletion at nadir. (A) Time course of viral load for increasing death rates of infected cells. CTL killing increases from red to light blue lines. As the lifespan of infected cells decreases (red to dark blue), peak viral load becomes lower, and the decay after the peak gets faster. However, for very high infected cell death rates (light blue) peak viral load decreases while the decay rate slows down. (B) Dependence of viral decay rate on peak viral load (black line) is nonmonotonic with positive correlation for high death rates of infected cells (yellow rectangle) and negative correlation for low death rates (blue rectangle). The arrows show the direction of increased death rate of infected cells. The black circle and the vertical dashed line mark the position of the maximum virus decay rate. (C) Depletion of CD4+ T cells at nadir is positively correlated to peak viral load. The black dot and the vertical dashed line mark the peak viral load corresponding to the maximal decay of virus. The horizontal line shows the CD4+ T cell depletion at nadir corresponding to the maximum decay after the viral peak (∼83%). We expect to see positive correlation between the viral peak and decay after the peak only CD4+ T cell depletion at nadir is lower than ∼83% (yellow rectangle). If CD4 depletion at nadir is higher (blue rectangle), we expect negative correlation between viral peak and decay.

Let us consider the consequences of CD8+ T cell cytolysis, when the course of the acute phase of infection in different animals varies only because of different infected cell lifespan. Figure 2A illustrates the expected relationship between peak viral load and viral decay rate, when virus is controlled by cytolysis of infected cells. When death rate of infected cells is sufficiently low, almost all CD4+ T cells are infected at nadir, so that the decay of virus is slow and almost exactly reflects the slow death rate of infected cells. Increasing the death rate (or killing by cytotoxic T lymphocytes) leads to increased observed decay rate while decreasing peak (red, pink, purple and dark blue lines in Figure 2A). However, when the infected cell death rate becomes very high, the overall level of infection is decreased sufficiently that the fraction of cells infected at peak is significantly reduced. This means that there are more uninfected cells available during the decay phase of virus, immediately after the peak. The observed rate of viral decay is the net effect of infected cell death balanced by the rate of new infections being produced. Thus, when the viral peak is sufficiently reduced, the decay rate slows down because there are progressively more uninfected cells available for infection during the decay phase (the light blue line in Figure 2A). This means that there must be a maximum decay rate for some intermediate peak viral load, and that the decay rate should become slower when the peak decreases further.

It is clear from the above reasoning that the correlation between peak viral load and viral decay rate can be either positive or negative. We would see a negative correlation when there are very few remaining uninfected cells, and faster decay is caused by faster disappearance of infected cells. In the positive correlation regime, the decay slows down with decreasing viral load because there are more uninfected cells at peak, thus allowing reinfection to balance the death of infected cells. Crucially, a positive correlation between peak viral load and observed viral decay can only occur when the number of remaining uninfected cells during decay is sufficiently large.

Our model (Eq.3–5) reproduces this behaviour. Figure 2B shows the generic dependence of the decay rate (Eq.9) on peak viral load (Eq.8) for increasing death rate of infected cells (*δ*) (in the direction of arrows), while Figure 2C shows the corresponding generic dependence of CD4+ T cell depletion at nadir (1-*T*_min_/*T*_0_) on peak viral load. Figures 2B and 2C were obtained using the method described in the Supplementary Material A. The viral decay rates in the blue rectangle in Figure 2B are negatively correlated to the peak, and correspond to more depleted CD4+ T cells in the blue rectangle in Figure 2C. The yellow rectangle in Figure 2B represents the observed negative correlation scenario, which occurs for better preserved CD4+ T cells in the yellow rectangle in Figure 2C.

The model predicts that the fastest viral decay rate (the maximum of the curve in Figure 2B) is always at the same level of depletion of CD4+ T cells (Figure 2C). The argument goes as follows: increased death rate of infected cells translates into the decrease of the reproductive ratio at the peak *R_P_*, which uniquely determines the position of the maximum decay rate in Figure 2B (see Supplementary Material A for demonstration). The maximum decay rate always occurs for *R_P_*≈2.15. For *R_P_*<2.15 (yellow rectangle), we expect a positive correlation between peak viral load and decay rate, and for *R_P_*>2.15 (blue rectangle) we expect a negative correlation.

Since CD4 depletion at nadir depends only on *R_P_* (Eq.7), the maximum level of depletion of CD4+ T cells that we expect to see with positive correlation between viral peak and decay corresponds to *R_P_*≈2.15, meaning that CD4 depletion should not exceed 83%.

**Figure 3:**
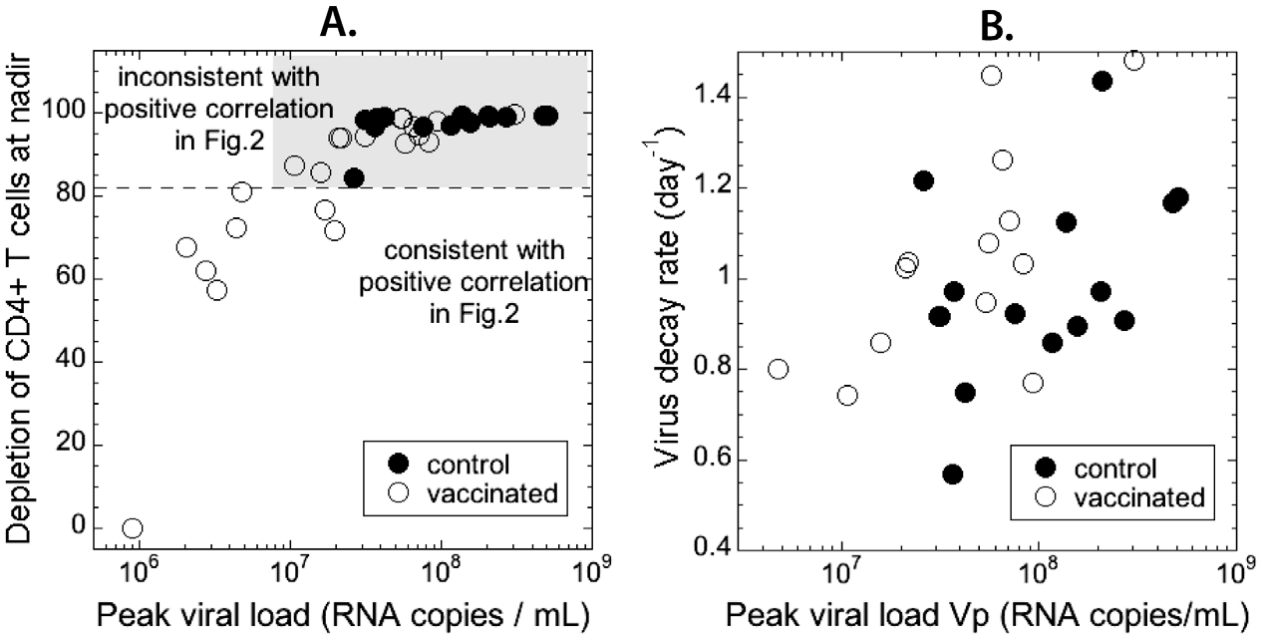
CD4+ T cell depletion at nadir. The observed depletion of CD4+ T cells at nadir is incompatible with the observed positive correlation between viral peak and decay if we assume that the CD8 response is cytolytic. (A) Dependence of nadir CD4+ depletion in control (full circles) and vaccinated (open circles) animals on peak viral load. Only the seven points below the dashed horizontal line (with CD4 depletion below 83%) are consistent with positive correlation in Figure 2. (B) The positive correlation between virus peak and decay remains even when the seven points with low CD4 depletion are removed.

SHIV_89.6P_ is a CXCR4-tropic virus that infects all CD4+ T cells. Therefore, if CD8+ T-cells are predominantly cytolytic and we have a positive correlation between peak viral load and viral decay, we do not expect maximum depletion of CD4+ T cells to be higher than 83%. The observed CD4+ T cell depletion at nadir in control and vaccinated animals is shown in Figure 3A against peak viral load. Only 7 vaccinated animals (out of 35) show depletion lower than 83% consistent with the positive correlation in Figure 1.

In order to determine if these seven animals are actually driving the positive correlation between viral peak and decay, we removed them from the data and recalculated the correlation for the remaining highly depleted animals. Surprisingly, the positive correlation was then even more significant (Spearman *r* = 0.465 and *p* = 0.0127).

### Effects of noncytolytic CD8+ T cell response

**Figure 4:**
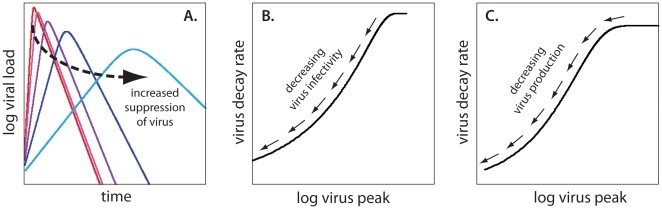
Noncytolytic immune response. Peak viral load and decay rate of virus are always positively correlated if virus suppression is due to noncytolytic immune response. (A) Decay rate of viral load after the peak stays almost constant (red, pink and purple lines) for high peak viremia, but slows down when virus is strongly suppressed (dark and light blue lines). (B) Viral decay rate increases with the increase in virus peak if CD8+ T cells control virus by decreasing infectivity. (C) A similar monotonic increase of decay rate with virus peak is observed assuming suppression of virus production.

Since the dynamics of viral decay and CD4+ T cell depletion are not consistently described by an immune response that results in increased killing of infected cells, we explored the consequences of noncytolytic activity of virus-specific CD8+ T cells. Some antiviral soluble factors released by CD8+ T cells suppress viral replication (by decreasing the probability of infection or the rate of virus production) without killing the infected cells [7]. The consequence is again the lowering of peak viral load. However, since the decay rate of virus after the peak reflects the balance of the death rate of infected cells (which is constant) and the rate of new infections, we see a fairly constant decay rate for high values of the peak, as in red, pink and purple lines in Figure 4A.

However, as the viral peak is further reduced, the proportion of uninfected cells persisting after the peak also increases considerably. This in turn has a consequence of leaving more uninfected cells available for infection after the peak. Again, since the observed decay rate is the net balance of the death rate of infected cells and the rate of new infections, we see slower decay as the peak is reduced (the light blue line in Figure 4A). In short, we expect peak viral load and virus decay rate to be always positively correlated. The dependence of virus decay on peak viral load for variable infectivity (Figure 4B) or for variable production of virus by infected cells (Figure 4C) in the model are similar and both predict a positive correlation between the peak and decay rate of viral load. The methods for obtaining the curves in Figure 4B and C are described in the Supplementary Material A.

### Comparing cytolytic and non-cytolytic effects

**Figure 5:**
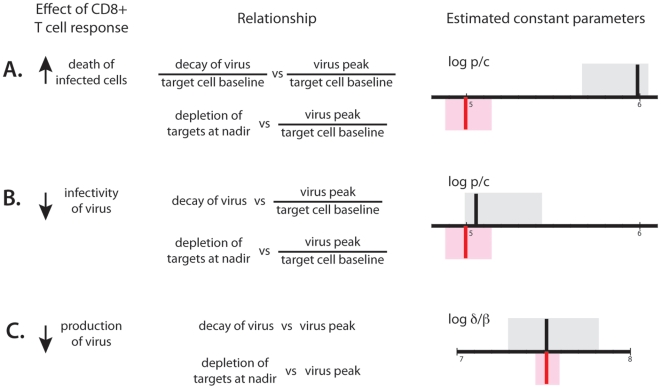
Fitting summary. Results of fitting the same parameter to the dependence of viral decay on peak (best estimate shown as black vertical line with 95% confidence intervals in grey) and to the dependence of CD4 depletion on viral peak (best estimate shown as red vertical line with 95% confidence intervals in pink) for different mechanisms of virus control. If we assume cytolytic control, the same parameter cannot simultaneously fit the two dependencies, but for noncytolytic mechanisms it can. A. The ratio of virus production to clearance *p/c* for cytolysis of infected cells; B. The ratio of virus production to clearance *p/c* for CD8 response decreasing infectivity, C. The ratio of infected cell death rate and infectivity *δ*/*β* if CD8 response decreases virus production, estimated from the dependence of virus decay and the dependence of CD4+ T cell depletion on peak viral load.

The analysis described above uses only the viral load kinetics in order to investigate whether the infection dynamics are consistent with a cytolytic or non-cytolytic model of infection. Here we found that a cytolytic model could result in either a positive or a negative relationship between peak viral load and viral decay rate, depending on the CD4+ T cell level. However, a non-cytolytic model consistently predicted a positive relationship. Since our experimental data also included the CD4+ T cell numbers for each animal, we extended our model to see if we would simultaneously account for both the viral and CD4+ T cell dynamics in using the cytolytic and non-cytolytic models. In other words, we assume that the differences in viral control among animals mainly arise from differences in the strength of CD8+ T cell effector function. The differences in CD8+ T cell function among animals can either result in differences in the death rate of infected cells (cytolytic model), or in the viral infectivity or viral production (non-cytolytic models), while the rest of the parameters vary among the animals in a random manner. The details of the fitting procedure are explained in the Supplementary Material B in Figures S1, S2, S3. The summary of the quantitative analysis is shown in Figure 5 and in Table 1.

**Table 1:** Results of nonlinear regression fitting of the same parameter from the dependence of virus decay and CD4+ nadir on virus peak, assuming different mechanisms of immune control.

Effect of CD8+ T cell response	Estimated parameter	Relationship	Best estimate (95% confidence interval)
Increase in *δ* (cytolysis)	*p*/*c*	Δ*_V_*/*T*_0_ vs. *V_P_*/*T*_0_	933.3 (467.7–1096.5) (copies/cell)×day^−1^
		1-*T*_min_/*T*_0_ vs. *V_P_*/*T*_0_	97.72 (75.86–151.4) (copies/cell)×day^−1^
Decreased *β* (noncytolytic)	*p*/*c*	Δ*_V_* vs. *V_P_*/*T*_0_	112.2 (85.1–263.0) (copies/cell)×day^−1^
		1-*T*_min_/*T*_0_ vs. *V_P_*/*T*_0_	97.72 (75.86–151.4) (copies/cell)×day^−1^
Decreased *p* (noncytolytic)	*δ*/*β*	Δ*_V_* vs. *V_P_*	3.24×10^7^ (2.00–6.46)×10^7^ (copies/mL)
		1-*T*_min_/*T*_0_ vs. *V_P_*	3.24×10^7^ (2.88–3.89)×10^7^ (copies/mL)

In Figure 5A and in Figure S1 we assumed that cytolysis of infected cells is the main mechanism of virus control, and determined the model parameter *p*/*c* that provided the best fit to the data. Using the data on the relationship between virus decay on virus peak, we first estimated the optimal *p/c* for this data (shown as a vertical black line with 95% confidence intervals (C.I.) in grey). We next estimated the optimal value of *p*/*c* that would fit the CD4+ T cell data (shown as a vertical red line with confidence intervals in pink). It is quite clear that a cytolytic model cannot simultaneously fit both the viral decay and CD4+ T cell data. This is because the observed depletion of CD4+ T cells at nadir is too high to be consistent with the positive correlation between viral peak and decay. The ratio of virus production to clearance estimated from the dependence of decay on peak is incompatible with the estimate from virus peak and CD4 nadir (the grey and the pink confidence intervals have no overlap).

Figures 5B and C show the results for two non-cytolytic mechanisms of control. In Figure 5B (and Figure S2) we fit the model of increasing immune response that partially blocks virus entry and thus limits the infectivity of the virus. Figure 5C (and Figure S3) shows the results of fitting the virus and CD4+ T cell dynamics when we assume that immune response suppresses virus production. In both cases, we estimated the best-fit parameters for the viral load data (black/grey) and CD4 T cells (red/pink). In each case, there is a large overlap in the confidence intervals of parameters estimated using the viral and CD4+ T cell data, demonstrating that the same parameters can simultaneously describe both viral and CD4+ T cell dynamics without any apparent contradictions.

Thus, our modelling shows that CD8+ T cell mechanisms that involve cytolysis of virus producing cells are incompatible with the experimental data, whereas non-cytolytic mechanisms are compatible, although we cannot differentiate whether reduced viral infectivity or reduced viral production by infected cells is the more likely mechanism.

### Cytolysis of infected cells before viral production

It has been recently shown [20], [21] that in SIV infection some epitopes from the Gag and Pol proteins are presented on MHC-I molecules as soon as 2 hours after viral entry into the infected cell, well before reverse transcription. Gag- and Pol-specific CD8+ T cells can recognize and eliminate infected cells in vitro almost immediately [20], [21]. Another study has shown that, after the start of production of new viral proteins (around 12 hours after infection of the cell), the presence of Nef downregulates the expression of MHC-I molecules [22], diminishing the probability of recognition by virus-specific CD8+ T cells. These effects would create a window period between 2–12 hours after cell infection, and before virion production, when infected cells can be recognized and killed most easily. If such “killing window” existed, what would be its impact on infection dynamics?

If infectivity of the virus remained unchanged, the number of uninfected target cells would remain the same as without any killing of infected cells. However, the number of infected cells producing virus would be diminished by the fraction that were killed before they started production. The viral replication would then appear the same as if virion production were reduced. The simplest model describing this effect would be the one where the Eq.4 is replaced by
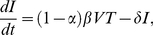
(1)where *α* is the fraction of infected cells killed in the window period. The solutions for target cells and viral load would then be the same as in the original model, but with viral production changed from *p* to (1-*α*)*p*. Thus, the conclusion is that our model of viral load behavior cannot distinguish between noncytolytic antiviral effects and infected cell killing in the window period between infection and the production of viral proteins.

## Discussion

After recognition of MHC-bound viral peptides, CD8+ T cells can kill infected cells and/or release into the environment a number of soluble factors that limit viral replication. We analysed the relationships of three main parameters of acute infection – peak viral load, decay rate of viral load after the peak and CD4+ T cell nadir, with the aim to identify the dominant mechanism of CD8+ T cell-mediated virus control in SHIV_89.6P_ infection that can consistently explain these three aspects of the acute phase dynamics.

We found that peak viral load and viral decay after the peak were positively correlated. Detailed analysis of the dynamics of decay of viral load showed that, if viral control is mediated by cytolysis of infected cells, the correlation between peak viral load and decay rate can be either positive or negative. However, the positive association can only occur when CD4+ T cell depletion is incomplete (less than ∼80%) (Figure 5C). While the experimental data show positive correlation between viral peak and decay, the massive depletion of CD4+ T cells at nadir is not consistent with the observed dynamics (Figure 3). Cytolytic control of SHIV_89.6P_ infection cannot simultaneously explain the observed relationships between peak viral load, decay of viral load after the peak and CD4+ T cell depletion at nadir.

If control of infection is mediated by noncytolytic mechanisms (such as suppression of new infections or virus production by infected cells), peak viral load and decay are expected to be always positively correlated as observed. Moreover, our modelling demonstrates that both the viral load and CD4+ T cell data are consistent with a non-cytolytic model.

One limitation of this study is the use of a CXCR4-tropic SHIV virus that may have different target cell specificity to the typical CCR5-tropism of HIV. The clear advantage of the SHIV model is that, since all CD4+ T cells can be infected, we were able to compare the dynamics of total CD4+ T cells with viral dynamics in individual hosts, by assuming that the virus infects all CD4+ phenotypes with similar probability, so that the CD4 depletion measured in blood reflects the depletion in other anatomical compartments like tissues and lymph nodes. By contrast, in infection with CCR5-tropic viruses, it is unclear which CD4+ T cell population is ideal to study in order to understand target cell availability [23]. However, the key question for this study is not which cells are infected, but how the CD8+ T cells control infection. It seems unlikely that the mechanisms of viral control by CD8+ T cells are completely different between CCR5-tropic and CXCR4-tropic viruses, since many of the epitopes targeted by CD8+ T cells are identical, and the viruses have a very similar decay rate under therapy (suggesting that the major virus-producing cells are behaving in a similar manner). Moreover, these results are consistent with recent results suggesting non-cytolytic control of SIV in vivo following depletion of CD8+ T cells and therapy during chronic infection [14], [15] and during immune escape [16]. Our study extends this work to suggest that viral control is also non-cytolytic during acute infection, and that vaccination does not modify this mechanism.

Our results demonstrate that the relationship between peak viral load, virus decay rate after the peak, and CD4+ T cell depletion cannot be simultaneously explained by a cytolytic mechanism of direct killing of productively infected cells by virus-specific CD8+ T cells. However, we cannot exclude the possibility that cytolysis of infected cells predominantly takes place in the window period after virus enters the cell, but before the start of production of viral proteins. On the other hand, non-cytolytic mechanisms of viral control involving reduced infectivity or reduced production of virus are consistent with the experimental data from SHIV vaccination analyzed here, as well as the results from CD8-depletion experiments and immune escape kinetics in SIV/SHIV-infected macaques ([14], [15], [16]), suggesting that further work is required to elucidate the cellular and molecular mechanisms by which vaccine-induced CD8+ T cells can control HIV infection.

## Methods

### Experimental data

In a previously published study [24], 35 rhesus macaques (*Macaca mulatta*) were challenged intravenously with 50% monkey infectious doses of X4-tropic SHIV_89.6P_ expressing SIV_mac239_ gag gene. Fourteen animals in this group were unvaccinated controls, while 21 were vaccinated with a variety of regimens, consisting of SIV gag-containing plasmid DNA (with different adjuvants), modified vaccinia Ankara, and adenovirus type 5 vectors, as previously reported. All animals were genotyped for the MHC class I Manu-A*01 allele presenting the immunodominant SIV gag epitope p11CM. All but one of the vaccinated animals and 6 out of 14 control animals were positive for this allele. The vaccinated animals were challenged at 6 weeks or at 12 weeks after the final boost. Viral loads and CD4+ T-cell counts were monitored in peripheral blood every 2 to 4 days until 4 weeks after infection and then weekly.

### Data analysis

We use experimental data for the acute peak viral load, the CD4+ T cell nadir and the maximum decay rate of the viral load after the peak. The peak viral load is the highest measured value of viral load during 4 weeks post infection and the target cell nadir is the lowest CD4+ T cell count within 10 days after the peak viral load. We define the exponential decay rate Δ*_V_* of viral load between two measurements at *t*_1_ and *t*_2_ as:
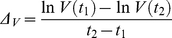
(2)


We calculate the decay rates for all the intervals for 2 weeks after the peak viral load, and use the maximum value. In all cases this maximum decay rate is calculated over time intervals of 3 or 4 days.

### Model

Mathematical models of viral dynamics have been successfully used to study HIV infection [25]. Here we adapt the standard model of viral dynamics [26], [27], [28], [29], using its reduced form [18], [19] to describe the behavior of uninfected (*T*) and infected (*I*) CD4+ T cells and viral load (*V*) in the acute SHIV_89.6P_ infection, where we have access to both viral load and CD4+ T cell number. The reduced standard model consists of equations
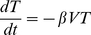
(3)

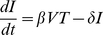
(4)

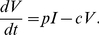
(5)


The infectivity *β* is the rate at which target cells become infected at unit virus concentration, and *δ* is the death rate of infected cells. Free virus is produced by infected cells at rate *p* and is cleared at rate *c*. The dynamics of replacement and death of uninfected target cells are important for their recovery after the nadir, but can be neglected in the description of infection between peak viral load and target cell nadir (where the rate of viral load decline is the fastest) [18], [26]. Thus Eqs.3–5 provide a good model of the correlations between viral peak and decay and target cell nadir in CXCR4-tropic infection.

In this model, cellular immune response can affect the death rate of infected cells *δ* (by killing of infected cells by cytotoxic T lymphocytes), infectivity *β* (by blocking viral entry) and production *p* (by suppression of virus production by infected cells).

We assume that infection-dependent parameters (*β*, *δ*, *p* and *c*) are approximately constant between peak viral load and the nadir of CD4+ T cells. Since the parameters in fact change in time because of change in immune response, this amounts to assuming that immune response does not change much during this period of approximately 10 days. If *T*_0_ is the baseline CD4 number, the course of acute infection in this period is characterized by the reproductive ratio at the peak *R_P_*, 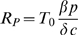
(6)


The reproductive ratio at the peak represents the number of infected cells generated by one infected cell during its lifetime, assuming the average level of immune response present between the peak and the nadir. It is a measure of virus replication in this period.

In the reduced standard model, the nadir of uninfected cells *T*_min_ is the solution of the equation [18]

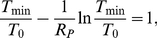
(7)


Peak viral load can be expressed as
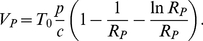
(8)


The fastest decay rate of viral load after the peak Δ*_V_* is at the nadir of CD4+ T cells [26],
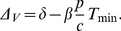
(9)


The reproductive ratio at the peak *R_P_* will differ among animals (e.g. with vaccination) because of the differences in the strength of their immune response, which translates into differences in some of the immunity-dependent parameters *δ*, *β*, or *p*. We shall analyse the predictions of these relationships assuming that the differences in viral control among animals mainly arise from differences in a single CD8+ T cell effector function, which either changes the lifespan of infected cells, or viral infectivity or production. In the model terms, this would translate into the interdependencies of viral peak and decay and target cell nadir, if the differences in *R_P_* among animals arise predominantly because of the differences in only one parameter (*δ* for cytolytic control or *β*, or *p* for noncytolytic effects). Using the model predictions, we shall determine which CD8 effector function best reproduces the observed viral and CD4+ T cell dynamics in the acute phase of infection.

## Supporting Information

Figure S1 Effects of cytolytic response. The ratio of viral production and clearance (p/c) cannot be consistently determined from experimental data for viral peak and decay and CD4 depletion if we assume cytolytic immune response. (A) Model prediction for dependence of decay rate on viral peak and the method for fitting to experimental data. The dependence is nonmonotonic with positive correlation for low peaks and negative correlation for higher peaks (black line). Increasing p/c shifts the curve in x-direction without changing its shape (red lines), while increasing the replicative capacity βp/c increases the maximum without shifting its position. (B) Best fit for p/c and βp/c from the dependence of virus decay on virus peak (both scaled by the baseline target cell number) is shown as black line. The envelope of confidence intervals for the two parameters is in grey. Because of the overall positive correlation between viral peak and decay, the best-fit p/c moves the position of maximum decay rate to the peak viral load higher than observed in most of the animals. Best fit (red line) and confidence intervals (pink) for replicative capacity when p/c is constrained to the best fit of peak – target nadir dependence. (C) Model prediction for dependence of CD4+ T cell depletion at nadir on viral peak and the method for fitting to experimental data. The basic shape of the dependence (black line) is parameter-independent and the increase in p/c shifts the curve in x-direction without changing its shape (red lines). (D) Best fit (red line) and confidence intervals (pink) for p/c determined from the dependence of CD4+ T cell depletion on peak viral load. Best fit and confidence intervals for p/c from peak – decay dependence are shown for comparison (black line and grey area respectively). Most data points lie on the left hand side of the curve in order to fit the negative correlation in (B). Red and black dashed lines in (B) and (D) show the peak viral load corresponding to the maximum viral decay rate for each fit.
(TIF)

Figure S2 Effects of reduced viral infectivity on the behaviour of viral load and CD4+ T cell depletion. The same ratio of virus production to clearance (*p*/*c*) fits the experimental data for the dependence of viral decay on peak and the dependence of CD4 depletion on viral peak if we assume that immune response limits virus infectivity. (A) Best fit for *p*/*c* and death rate of infected cells (*δ*) from the dependence of virus decay on virus peak (scaled by CD4+ T cell number) is shown as black line. The envelope of confidence intervals for the two parameters is in grey. Best fit (red line) and confidence intervals (pink) for *δ* when *p*/*c* is constrained to the best fit of dependence CD4+ nadir on virus peak. (B) Best fit (red line) and confidence intervals (pink) for *p*/*c* determined from the dependence of CD4+ T cell depletion on peak viral load (scaled by baseline target cell number). Best fit and confidence intervals for *p*/*c* from peak – decay dependence are shown for comparison (black line and grey area respectively).
(TIF)

Figure S3 Effects of decreased virus production on the behaviour of viral load and CD4+ T cell depletion. The same ratio of infected cells death rate to infectivity (*δ*/*β*) fits the experimental data for the dependence of viral decay on peak and the dependence of CD4 depletion on viral peak if we assume that immune response suppresses virus production rate. (A) Best fit for *δ*/*β* and infected cells death rate (*δ*) from the dependence of virus decay on virus is shown as black line. The envelope of confidence intervals for the two parameters is in grey. Best fit (red line) and confidence intervals (pink) for *δ* when *β*/*δ* is constrained to the best fit of dependence CD4+ nadir on virus peak. (B) Best fit (red line) and confidence intervals (pink) for *β*/*δ* determined from the dependence of CD4+ T cell depletion on peak viral load. Best fit and confidence intervals for *β*/*δ* from peak – decay dependence are shown for comparison (black line and grey area respectively).
(TIF)
