# Supplementary figures and images for: Vaccination-Induced Noncytolytic Effects in the Acute Phase of SHIV Infection
Source: PLoS One. 2010 Nov 30;5(11):e15083. doi: 10.1371/journal.pone.0015083 (PMC2994900; doi:10.1371/journal.pone.0015083)

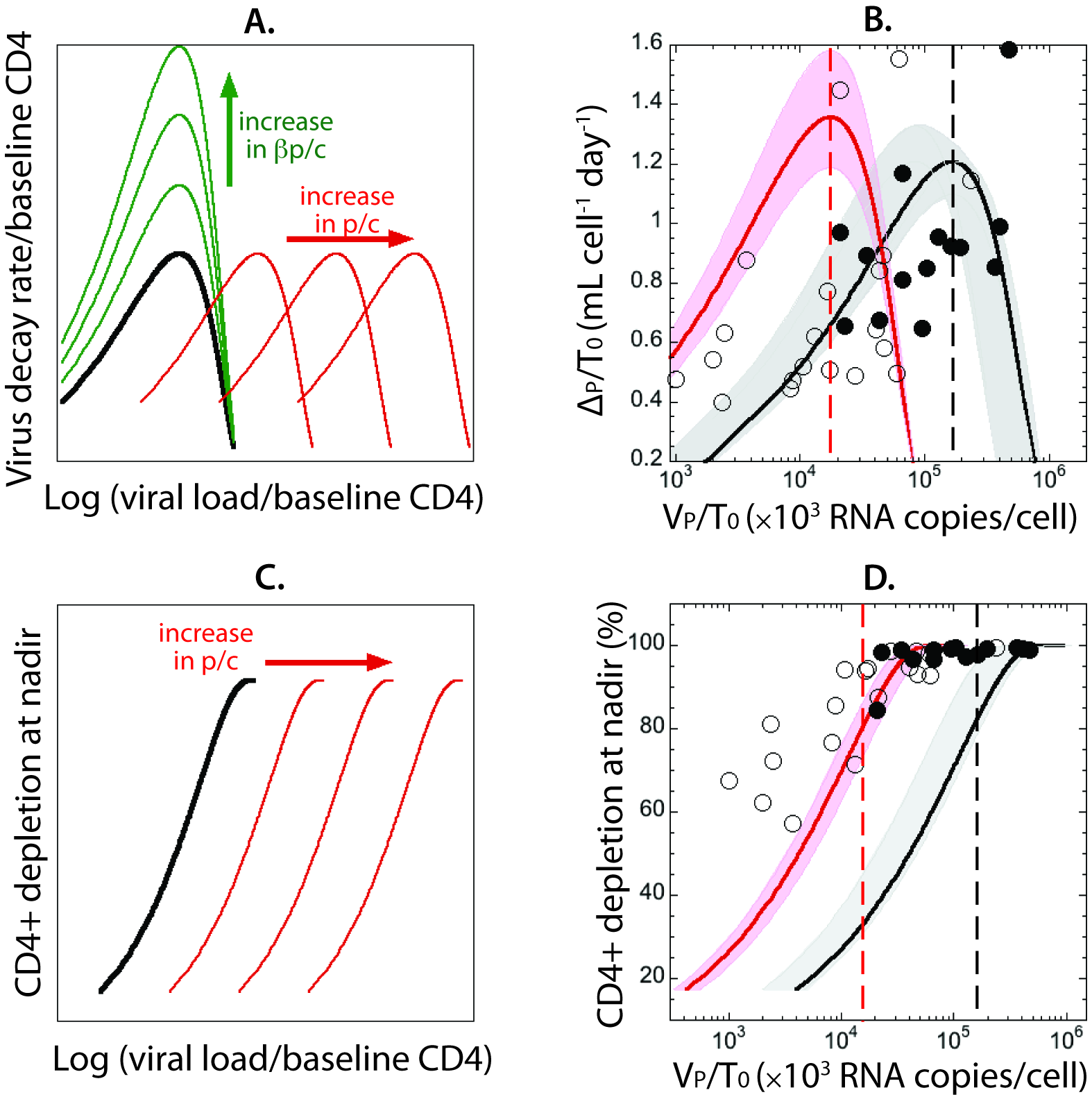

Supplement: Figure S1 — Effects of cytolytic response. The ratio of viral production and clearance (p/c) cannot be consistently determined from experimental data for viral peak and decay and CD4 depletion if we assume cytolytic immune response. (A) Model prediction for dependence of decay rate on viral peak and the method for fitting to experimental data. The dependence is nonmonotonic with positive correlation for low peaks and negative correlation for higher peaks (black line). Increasing p/c shifts the curve in x-direction without changing its shape (red lines), while increasing the replicative capacity βp/c increases the maximum without shifting its position. (B) Best fit for p/c and βp/c from the dependence of virus decay on virus peak (both scaled by the baseline target cell number) is shown as black line. The envelope of confidence intervals for the two parameters is in grey. Because of the overall positive correlation between viral peak and decay, the best-fit p/c moves the position of maximum decay rate to the peak viral load higher than observed in most of the animals. Best fit (red line) and confidence intervals (pink) for replicative capacity when p/c is constrained to the best fit of peak – target nadir dependence. (C) Model prediction for dependence of CD4+ T cell depletion at nadir on viral peak and the method for fitting to experimental data. The basic shape of the dependence (black line) is parameter-independent and the increase in p/c shifts the curve in x-direction without changing its shape (red lines). (D) Best fit (red line) and confidence intervals (pink) for p/c determined from the dependence of CD4+ T cell depletion on peak viral load. Best fit and confidence intervals for p/c from peak – decay dependence are shown for comparison (black line and grey area respectively). Most data points lie on the left hand side of the curve in order to fit the negative correlation in (B). Red and black dashed lines in (B) and (D) show the peak viral load corresponding to the maxim [file pone.0015083.s001.tif]

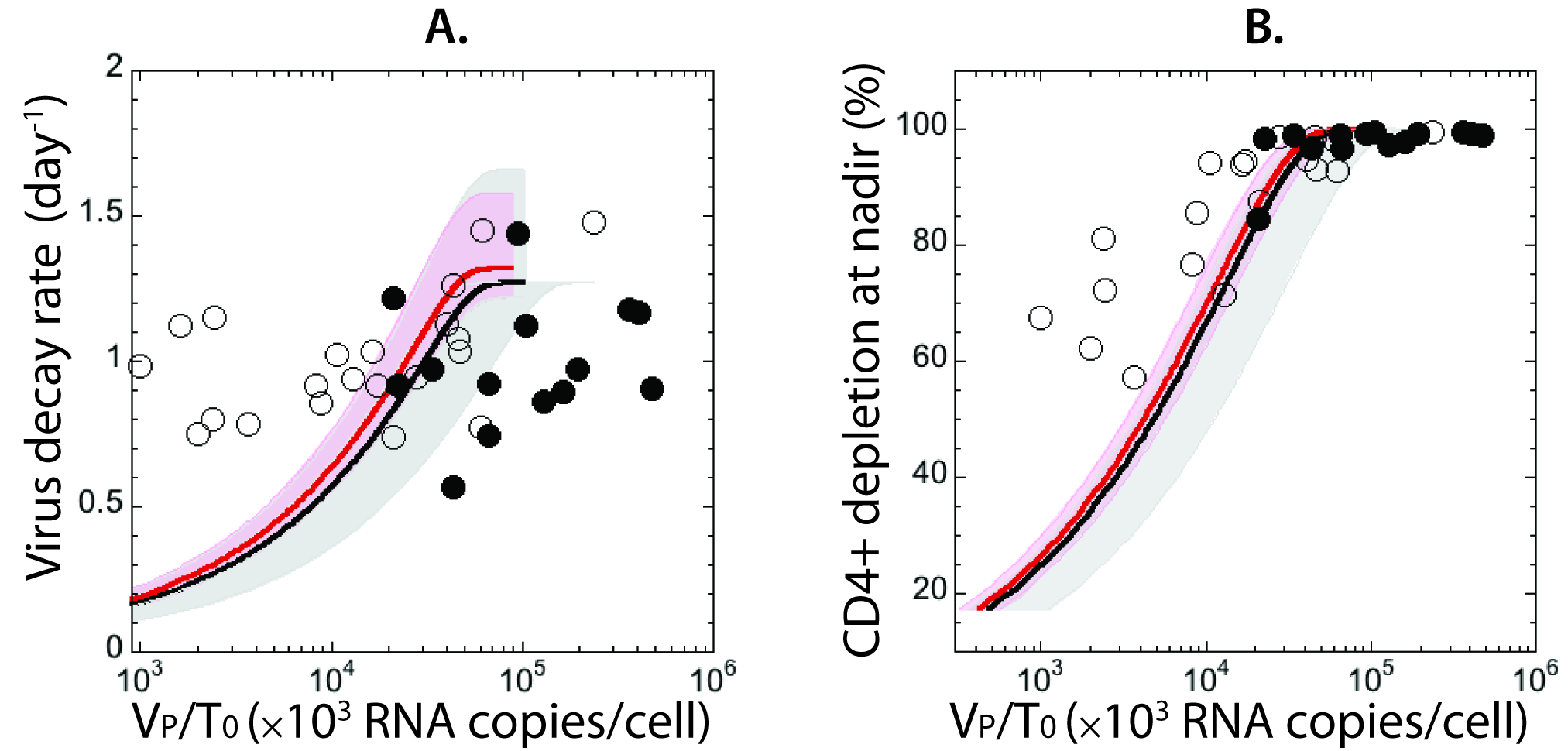

Supplement: Figure S2 — Effects of reduced viral infectivity on the behaviour of viral load and CD4+ T cell depletion. The same ratio of virus production to clearance (p/c) fits the experimental data for the dependence of viral decay on peak and the dependence of CD4 depletion on viral peak if we assume that immune response limits virus infectivity. (A) Best fit for p/c and death rate of infected cells (δ) from the dependence of virus decay on virus peak (scaled by CD4+ T cell number) is shown as black line. The envelope of confidence intervals for the two parameters is in grey. Best fit (red line) and confidence intervals (pink) for δ when p/c is constrained to the best fit of dependence CD4+ nadir on virus peak. (B) Best fit (red line) and confidence intervals (pink) for p/c determined from the dependence of CD4+ T cell depletion on peak viral load (scaled by baseline target cell number). Best fit and confidence intervals for p/c from peak – decay dependence are shown for comparison (black line and grey area respectively). (TIF) [file pone.0015083.s002.tif]

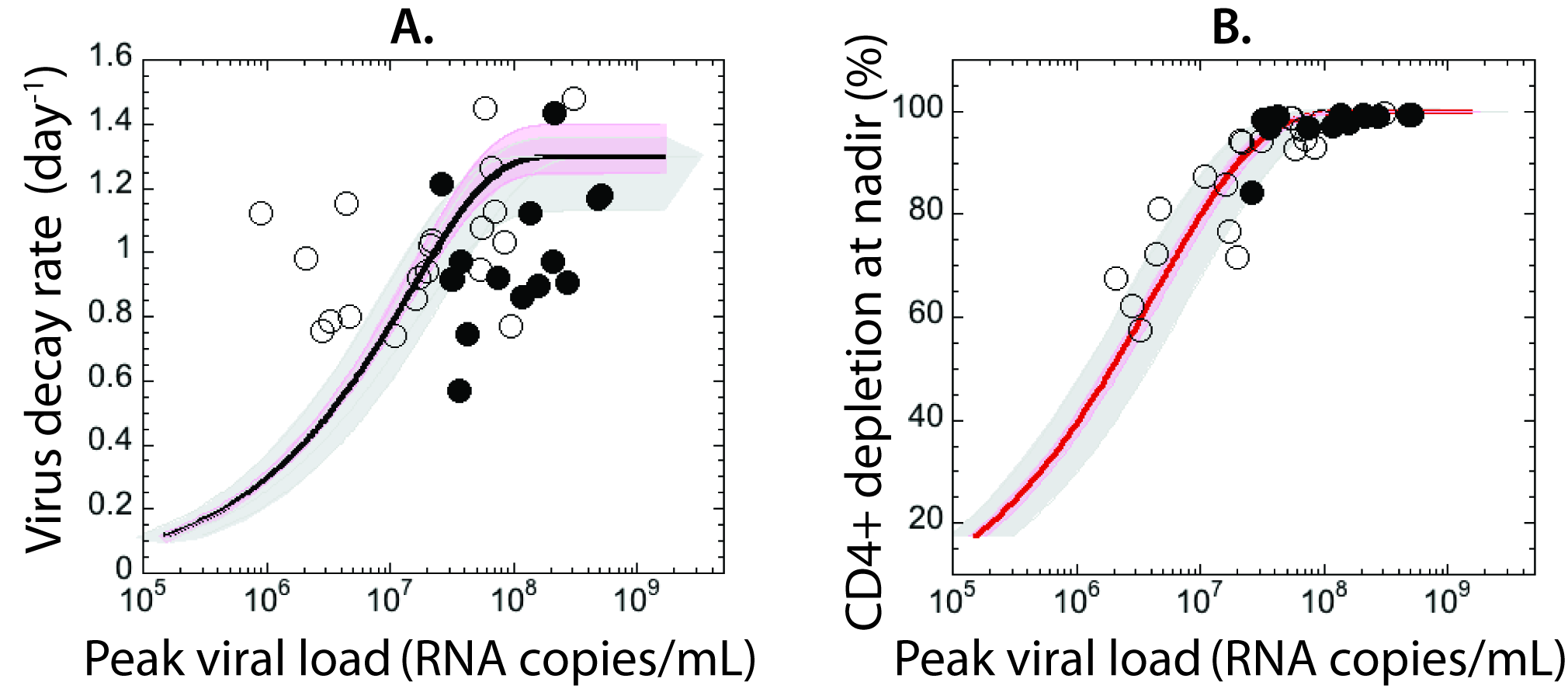

Supplement: Figure S3 — Effects of decreased virus production on the behaviour of viral load and CD4+ T cell depletion. The same ratio of infected cells death rate to infectivity (δ/β) fits the experimental data for the dependence of viral decay on peak and the dependence of CD4 depletion on viral peak if we assume that immune response suppresses virus production rate. (A) Best fit for δ/β and infected cells death rate (δ) from the dependence of virus decay on virus is shown as black line. The envelope of confidence intervals for the two parameters is in grey. Best fit (red line) and confidence intervals (pink) for δ when β/δ is constrained to the best fit of dependence CD4+ nadir on virus peak. (B) Best fit (red line) and confidence intervals (pink) for β/δ determined from the dependence of CD4+ T cell depletion on peak viral load. Best fit and confidence intervals for β/δ from peak – decay dependence are shown for comparison (black line and grey area respectively). (TIF) [file pone.0015083.s003.tif]
